# Adaptation and assessments of the Chinese version of the ICECAP-A measurement

**DOI:** 10.1186/s12955-018-0865-3

**Published:** 2018-03-12

**Authors:** Chengxiang Tang, Yao Xiong, Hongyan Wu, Judy Xu

**Affiliations:** 1 School of Public Administration, Guangzhou University, Guangzhou, China; 2 School of Public Administration, Southwestern University of Finance and Economics, 555# LiuTai Rd, Wenjiang District, Chengdu, Sichuan 610072 China; 3 School of Medicine and Health Management, Guizhou Medical University, Guiyang, China

**Keywords:** ICECAP-A, Capability, Validation, Assessment, China

## Abstract

**Background:**

This study adapts the ICECAP measure for Adults (ICECAP-A) to assess its capacity to measure the quality of life in China for economic evaluation.

**Methods:**

Qualitative and quantitative methods were used to translate the ICECAP-A measure for wellbeing, established by the University of Birmingham, UK, to the Chinese cultural context. A focus group discussion solved the appropriateness and wording of the ICECAP attributes in Chinese; and a randomly selected sample of 1000 adults aged over 18 years were online surveyed. We conducted psychometric tests and compared the factors influencing the ICECAP-A measure with those influencing EQ-5D-3 L.

**Results:**

Members of the focus group discussion agreed that the five attributes of the ICECAP-A measure are sufficient to evaluate wellbeing in China. However, the terms “being settled” and “friendship” were changed to “stability” and “kindness” for the Chinese cultural context. Our results show that the Chinese version of ICECAP-A has good internal consistency with an overall Cronbach’s Alpha coefficient of 0.7999. The concurrent validity indicates that ICECAP-A is moderately correlated with EQ-5D-3 L (r ≤ 0.52).

**Conclusions:**

The ICECAP-A measure can be adapted to evaluate wellbeing in China, but cultural changes to the wording are necessary. It is a valid measurement of wellbeing and can complement the EQ-5D already used in China. However, further work is still needed to evaluate the sensitivity of the ICECAP-A measure in relation to public health and social care.

## Background

Economic evaluations for health technology or healthcare services have been employed by decision-makers to overcome the challenges of rising health care expenditure in the context of rapidly developing medical innovations [1–3]. Beyond all costs measurement, outcome measures that can capture the broader impacts and multiple dimensions of these healthcare interventions are critical for economic evaluation. Moreover, economic evaluations are going beyond clinical medical services, to include public health, social care interventions, and even more general public policies and regulations [4, 5]. Therefore, it is becoming more important and challenging to seek for an approach capturing individuals’ benefits as well as their well-beings in a more general conceptual framework.

Currently, the quality adjusted life year (QALY) is a widely accepted instrument of measurement in health economic evaluation [6]. However, the traditional framework of measuring quality of life (QoL) for QALYs focuses only on health-related QoL and may not be able to capture multi-dimensional outcomes which are important to quality of life in general [7, 8]. Alternative measures based on Amartya Sen’s capability approach are starting to become available [9–11]. The capability approach is primarily developed to assess an individual’s advantage based on a person’s ability to achieve ‘functionings’ in life that are valuable to them. Yet, health service researchers and bioethicists have considered Sen’s capability approach as a way to provide a more comprehensive well-being picture of individuals than preference-based HRQoL measures, such as QALYs.

One option for measuring capability wellbeing is the suite of ICECAP measures [12–14]; these have been developed in recent years to measure more general wellbeing than the traditional framework of health-related quality of life (HRQoL) permits. The ICECAP-A is a measure specially designed for adults. It was generated based on interviews with adults in the general population and consists of five attributes: Stability, Attachment, Autonomy, Achievement and Enjoyment [12]. Studies suggest that these attributes capture individuals’ freedoms to function, as well as potentially key outcomes of public health policies and interventions [12, 14].

So far, most evidence on the use of this measure relates to its original UK version. This version includes studies of the measure’s content validity and acceptability [15, 16], its construct validity in both general population and patient groups [17–19], its reliability [20] and its sensitivity to change [18, 21]. The relationship of the ICECAP-A to the EQ-5D measure of HRQoL has also been explored [22, 23]. As of January 2017, the ICECAP questionnaire has been translated into seven other languages (Chinese, Dutch, French, German, Spanish, Turkish and Welsh) and validated in Dutch [24]. However, a psychometrical analysis of a version translated for a substantially different culture has not been undertaken.

In this study, we used both qualitative and quantitative methods to develop a Chinese translation of the ICECAP-A instrument and validate it. We compared the Chinese version of ICECAP-A with the Chinese EQ-5D to identify similarities and differences between health-related and general quality of life measures. This study is the first to explore the use of a capability approach in the health field in China and the first to use any of the ICECAP measures in this context. This paper aims to introduce this new instrument for measuring capability for well-being to health economists and decision-makers in China.

## Methods

A series of qualitative and quantitative analysis were conducted in 2016 to develop a translation of the ICECAP-A into Chinese and to validate this translated ICECAP-A version. Ethics approval for the work was obtained from the Ethics Review Committee at the Affiliated Zhongshan Hospital of Xiamen University.

### ICECAP-A translation and cross-cultural adaptation

The research team translated the ICECAP-A into Chinese with permission from the ICECAP team at the Institute of Applied Health Research at the University of Birmingham. Initially, two team members translated the original version of the ICECAP-A questionnaire into Chinese. A focus group of four experts and eight team members then evaluated the appropriateness of the five translated attributes of ICECAP-A for a Chinese context during a half-day meeting. The experts were researchers with backgrounds in the social sciences, and the team members were postgraduate students in the health services and policy fields. A moderator was responsible for hosting and tape-recording the discussion. The main issues discussed were the factors that they considered important for their own wellbeing and the translational appropriateness of the five attributes based on the Chinese ICECAP-A questionnaire.

After the panel discussion, the revised questionnaire was pilot-tested with a convenient sample of 25 undergraduate students. They were asked to fill in both ICECAP-A and EQ-5D questionnaires and provide feedback and comments on the wording of the questions concurrently. Five community healthcare providers were asked to undertake the same process and provide feedback by phone or email.

We also selected two persons for back translation: one English native speaker who can speak and write Chinese very well, and one Chinese native speaker who is a senior English instructor. They were not aware of the concepts being explored before the translation. Both versions of the back translation were later merged, based on the original format of ICECAP questionnaire produced by University of Birmingham. Another two Chinese-speaking students were also asked for a back translation. Both of them can read English fluently and were involved in the earlier focus group discussion, they were also aware of the Chinese version of the questionnaire. Their back translation was used to double-check the cultural differences in the language translation.

### An online survey

An online survey was administered to Chinese adults over 18 years of age. The survey was conducted through SoJump, which is the largest Internet market research firm in China. A sample of 1127 respondents was randomly selected from its 2.6 million registered subjects (http://ctang1.sojump.com/jq/8427388.aspx). Upon completion of the survey, respondents received points that can later be exchanged for cash. This is not a representative sample of the general Chinese population, but can be a representative sample of adult netizens. Detailed information about the panel can be found at the website at http://www.sojump.com/. Other recent studies in China have used the same online approach [25, 26]. In addition to asking participants to complete ICECAP-A, they were also asked to complete the EQ-5D-3 L and to provide socioeconomic information including: information about education, marital status, employment, health insurance, income, and housing. After deleting the results of those who finished the questionnaire in less than 2 min and those with missing or multiple responses, the final sample contained 975 valid responses. Table 1 shows the definition of the key variables.

**Table 1:** Definition of key variables

Characteristics	Variables	Type	Definition
Socioeconomics			
Age	Age	continuous variable	Respondents age
Gender	Female	dummy variable	Female is 1, male is 0
Education	High school degree or below	dummy variable	High school degree or below is 1, the other is 0
	College degree	dummy variable	College degree is 1, the other is 0
	Bachelor degree or above	dummy variable	Bachelor degree or above is 1, the other is 0
Marital status	Unmarried	dummy variable	Unmarried is 1, the other is 0
	Married and cohabiting	dummy variable	Married and cohabiting is 1, the other is 0
	Other marital status	dummy variable	Other marital state is 1, the other is 0
Job	Business services	dummy variable	Business services is 1, the other is 0
	Administration	dummy variable	Administration is 1, the other is 0
	Service personnel	dummy variable	Service personnel is 1, the other is 0
	Technical personnel	dummy variable	Technical personnel is 1, the other is 0
	Other job	dummy variable	Other job is 1, the other is 0
Household registration	Rural	dummy variable	Rural is 1, non-rural is 0
Health insurance	Full Insurance	dummy variable	Full Insurance is 1, the other is 0
	Urban Workers’ Basic Medical Insurance	dummy variable	Urban Workers’ Basic Medical Insurance is 1, the other is 0
	Urban Residents Basic Medical Insurance	dummy variable	Urban Residents Basic Medical Insurance is 1, the other is 0
	New Rural Cooperative Medical Insurance	dummy variable	New Rural Cooperative Medical Insurance is 1, the other is 0
	Commercial Medical Insurance	dummy variable	Commercial Medical Insurance is 1, the other is 0
	Other insurance	dummy variable	Other insurance is 1, the other is 0
	No insurance	dummy variable	No insurance is 1, the other is 0
Incomes adults	Incomes adults	continuous variable	The number of adults who have incomes
Monthly income per capita	<¥1000	dummy variable	The monthly income less than ¥1000 is 1, the other is 0
	¥1000 to ¥2000	dummy variable	The monthly income between ¥1000 and ¥2000 is 1, the other is 0
	¥2000 to ¥3000	dummy variable	The monthly income between ¥2000 and ¥3000 is 1, the other is 0
	¥4000 to ¥5000	dummy variable	The monthly income between ¥4000 and ¥5000 is 1, the other is 0
	More than ¥5000	dummy variable	The monthly income more than 5000 yuan is 1, the other is 0
	Unknown monthly income	dummy variable	The unknown monthly income is 1, the other is 0
Income source	Salary	dummy variable	Salary of income source is 1, the other is 0
	Agriculture production or business	dummy variable	Agriculture production or business of income source is 1, the other is 0
	Other income source	dummy variable	Other income source is 1, the other is 0
Number of houses	Number of houses	continuous variable	The number of houses
Quality of Life			
ICECAP-A	ICECAP-A score	continuous variable	The score of ICECAP, and the value range is 0 to 1
EQ-5D-3 L	EQ-5D-3 L score	continuous variable	The score of EQ-5D, and the value range is −0.147 to 1
EQ-VAS	EQ-VAS	continuous variable	The score of EQ-VAS, and the value range is 0 to 100

### Psychometric tests

1. Acceptability

Data from the focus group discussion, pilot-testing and the online survey were used to assess acceptability. Specifically, we used information about the time taken to answer the ICECAP-A questionnaire and those with missing or multiple responses to analyse the acceptability of the responses.

2) Reliability

The reliability for the questionnaire as a system can be tested to check the reliability of the ICECAP-A where Cronbach’s alpha, with a value of > 0.70 is considered acceptable.

3) Validity

Exploratory factor analysis (EFA) was conducted for both the EQ-5D and ICECAP-A. We also conducted Polychoric correlation analysis between the scores for the ICECAP-A, EQ-5D-3 L, and EQ-VAS. We employed Polychoric correlation analysis instead of Pearson correlation, because the former is employed when the measurement of variables is based on an ordinal scale. Data from the online survey was also used to analyse the relationships between ICECAP-A scores and other socio-economic statuses determined by descriptive statistics and multivariable linear regression models. The different factors that influence the ICECAP-A and EQ-5D-3 L were identified.

In the study, the overall ICECAP score was calculated using the UK value set [27]. We used the EQ-5D-3 L utility values set that were developed recently, based on the Chinese population [28]. Individual attributes for the ICECAP-A questionnaire ranged from the highest capability level (4) to the lowest (1), whereas each dimension of the EQ-5D questionnaire ranges from the highest level (1) to the lowest (3). Therefore, the highest capability score is (4,4,4,4,4) whilst the highest EQ-5D-3 L score is (1,1,1,1,1).

## Results

### Online-survey description

Table 2 shows the descriptive statistics for the sample and Table 3 shows that the modal response to ICECAP-A for all attributes was at the second best level ranging from 51% for autonomy (independence) to 63% for attachment (love, kindness and support). The modal response for EQ-5D-3 L was at the top level for all dimensions ranging from 69% for anxiety/depression to 94% for self-care.

**Table 2:** Sample characteristics (*n* = 975)

Characteristics	Category	Frequency	Percentage(%)
Socioeconomics			
Age	18 ~ 29	352	36.1
[Mean: 33.66, SD: 8.6483]	30 ~ 44	509	52.2
	45 ~ 64	100	10.3
	> 64	14	1.4
Gender	Female	513	52.6
	Male	462	47.4
Education	High school degree or below	77	7.9
	College degree	223	22.9
	Bachelor degree or above	675	69.2
Marital status	Unmarried	205	21.0
	Married and cohabiting	734	75.3
	Other marital status	36	3.7
Job	Other job	177	18.2
	Business services	73	7.5
	Administration	270	27.7
	Service personnel	154	15.8
	Technical personnel	301	30.9
Household registration	Non rural	811	83.2
	Rural	164	16.8
Health insurance	No insurance	33	3.4
	Full Insurance	190	19.5
	Urban Workers’ Basic Medical Insurance	676	69.3
	Urban Resident Basic Medical Insurance	267	27.4
	New Rural Cooperative Medical Insurance	107	11.0
	Commercial Medical Insurance	214	21.9
	Other insurance	8	0.8
Incomes adults [Mean: 2.4308, SD: 0.8037]			
Monthly income per capita	<¥1000	6	0.6
	¥1000 ~ ¥2000	41	4.2
	¥2000 ~ ¥3000	194	19.9
	¥4000~¥5000	627	64.3
	≥¥5000	101	10.4
	Unknown monthly income	6	0.6
Income source	Salary	928	95.2
	Agriculture production or business	156	16.0
	Other income source	160	16.4
Number of houses	1~ 5	782	80.2
[Mean: 4.3538, SD: 5.0288]	6~ 10	180	18.5
	11~ 20	11	1.1
	≥21	2	0.2
Quality of Life			
ICECAP-A	< 0.5	13	1.3
[Mean: 0.8481, SD: 0.1152]	0.5~ 0.75	124	12.7
	0.75~ 1	838	85.9
EQ-5D-3L^a^	< 0.5	7	0.7
[Mean: 0.8920, SD: 0.1151]	0.5~ 0.75	83	8.5
	0.75~ 1	885	90.8
EQ-VAS [Mean: 77.57, SD: 17.576]			

^a^The score of EQ-5D-3 L was used the value set based on the Chinese population (Liu et al., 2014)

**Table 3:** Response to ICECAP-A & EQ-5D-3 L questionnaire (*n* = 975)

Attribute	Percentage(%)
ICECAP-A	
Sense of stability and security	
I have the sense of stability and security in all aspects of my life.	28.51
I have the sense of stability and security in most aspects of my life.	62.36
I have the sense of stability and security in a few aspects of my life.	8.51
I do not have the sense of stability and security in any aspect of my life.	0.62
Love, kindness and support	
I can get a great amount of love, kindness and support.	28.92
I can get much love, kindness and support.	62.87
I can get a little love, kindness and support.	8.10
I can not get any love, kindness and support.	0.10
Independence	
I am able to be fully independent.	40.62
I am able to be independent in many aspects.	50.67
I am able to be independent in some aspects.	8.31
I am unable to be independent at all.	0.41
Achievement and progress	
I can make achievement and progress in all aspects of my life.	20.82
I can make achievement and progress in many aspects of my life.	53.44
I can make achievement and progress in some aspects of my life.	24.82
I can not make achievement and progress in any aspect of my life.	0.92
Enjoyment and pleasure	
I enjoy my own life very much and can get plenty of joy from it.	31.90
I enjoy my own life and can get some joy from it.	61.23
I barely enjoy my own life and can only get a little joy from it.	6.46
I do not enjoy my life at all and can not get any joy from it	0.41
EQ-5D-3 L	
Mobility	
No problems	93.23
Moderate problems	6.67
Severe problems	0.10
Self-care	
No problems	94.26
Moderate problems	5.33
Severe problems	0.41
Usual activities	
No problems	92.82
Moderate problems	6.67
Severe problems	0.51
Pain/Discomfort	
No problems	77.54
Moderate problems	21.85
Severe problems	0.62
Anxiety/Depression	
No problems	68.72
Moderate problems	30.56
Severe problems	0.72

### Psychometric tests

1. Acceptability

In general, the translated Chinese version of ICECAP-A can be understood and self-completed by members of the general adult population in around 5 min. Participants in the discussion group, field tests, and health providers agree that the five attributes contained in the translated ICECAP-A are sufficient to evaluate well-being in China. Three slight modifications were deemed useful to adapt the ICECAP-A to a Chinese cultural context. First, respondents felt uncomfortable when talking about “love/friendship” because it is not a word commonly used in their daily life. Consequently, we chose the closest Chinese word, “kindness”. Second, “feel settled” has the same meaning as “stability” when translated, but it is longer and was considered less acceptable. So “stability” was used instead of “feel settled”. Finally, we added an introduction to the questionnaire to guide participants. It stated:

“The following are questions related to your quality of life in general. Please take your own capabilities and external environment into consideration while answering each question. For example, a question like “sense of stability and security” is related to your health condition, working condition, family economic condition and social relationships. It may also require your judgment regarding whether or not it is in a harmonious and stable state”.

Both the revised Chinese version of ICECAP-A and the back-translated version were approved by the ICECAP team in the University of Birmingham.

2) Reliability

As shown in Tables 4 and 5, each item of the ICECAP-A and EQ-5D-3 L in Chinese is independent as the correlation factor ranges from 0.2 to 0.6. Moreover, both the Cronbach’s Alpha coefficient are more than 0.7, which suggests an appropriate level of reliability.

3) Validity

**Table 4:** Correlation between each item in ICECAP-A

	Stability	Attachment	Autonomy	Achievement	Enjoyment
Stability	1.0000	–	–	–	–
Attachment	0.5589	1.0000	–	–	–
Autonomy	0.3417	0.3790	1.0000	–	–
Achievement	0.5246	0.4903	0.3883	1.0000	–
Enjoyment	0.4519	0.5028	0.3684	0.4780	1.0000
Cronbach’s Alpha	0.7999				

^*^All coefficients are significant at the 1% level

**Table 5:** Correlation between each item in EQ-5D-3 L

	Mobility	Self-care	Usual activities	Pain/discomfort	Anxiety/depressed
Mobility	1.0000	–	–	–	–
Self-care	0.5886	1.0000	–	–	–
Usual activities	0.5298	0.5938	1.0000	–	–
Pain/discomfort	0.3897	0.3406	0.3856	1.0000	–
Anxiety/depressed	0.2615	0.2305	0.2391	0.4448	1.0000
Cronbach’s Alpha	0.7245				

^*^All coefficients are significant at the 1% level

**Fig. 1:**
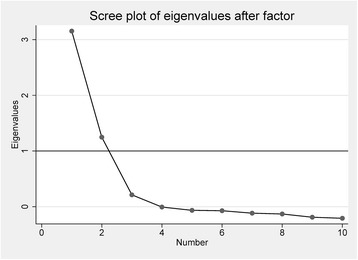
Factors eigenvalue of variance in the ICECAP-A and EQ-5D-3 L

Table 6 shows KMO (Kaiser-Meyer-Olkin) values for ICECAP, EQ-5D-3 L, the variables of which appear to warrant an exploratory factor analysis. In general, the items are meritorious in the ICECAP-A (0.85–0.91) and middling in the EQ-5D-3 L (0.78–0.86), indicating that performing an exploratory factor analysis is worthwhile. In Fig. 1 a scree plot presents a graph of eigen values, through the number of eigen values greater than one, a two factor solution was found to be optimal.

**Table 6:** KMO values of ICECAP-A and EQ-5D-3 L

Variable	KMO
ICECAP-A	
Stability	0.85
Attachment	0.85
Autonomy	0.91
Achievement	0.85
Enjoyment	0.88
EQ-5D-3 L	
Mobility	0.82
Self-care	0.78
Usual activities	0.81
Pain/ discomfort	0.86
Anxiety/ depressed	0.86
Overall	0.84

Table 7 presents the principal results of the exploratory factor analysis: the majority of ICECAP-A items loaded onto Factor 1, and the majority of EQ-5D-3 L items (Mobility, Self-care, Usual Activities and Pain/ discomfort) loaded strongly onto Factor 2, while the item of Anxiety/ depression loaded strongly onto Factor 1. The factor correlation is 0.4, meaning the promax rotation was an appropriate choice for the analysis. The results indicated that there is a different construct between the ICECAP-A and EQ-5D-3 L, providing different information. They also show certain similarity to results presented by Davis et al. [29] using the ICECAP-O and Keeley et al. [22] to analyse patients with knee pain [22, 30].

**Table 7:** Exploratory factor analysis comparing the ICECAP-A and EQ-5D (*n* = 975)

	Rotated promax	
	Factor 1	Factor 2
ICECAP-A		
Stability	**0.73**	−0.04
Attachment	**0.74**	−0.07
Autonomy	**0.46**	0.16
Achievement	**0.72**	−0.10
Enjoyment	**0.67**	− 0.01
EQ-5D-3 L		
Mobility	−0.05	**0.74**
Self-care	−0.09	**0.77**
Usual activities	−0.07	**0.74**
Pain/ discomfort	0.21	**0.46**
Anxiety/ depressed	**0.38**	0.25
Factor correlation(s)	0.4	

Table 8 shows the correlation coefficients of each item and total scores of ICECAP-A, EQ-5D-3 L, and EQ-VAS. In general, the item correlation between the two instruments is weak (Polychoric’s r 0.24–0.52). Stability, attachment, achievement, enjoyment in ICECAP-A have a higher correlation with anxiety/depression than other items in EQ-5D-3 L (Polychoric’s r 0.36–0.51), but the autonomy in ICECAP-A has a higher correlation with mobility and, self-care in EQ-5D-3 L (Polychoric’s r 0.52). However, each item in the ICECAP-A has a weak correlation with the EQ-5D-3 L and EQ-VAS scores (Polyserial’s r 0.27–0.39), and each item in the EQ-5D-3 L has a moderate correlation with the ICECAP-A score (Polyserial’s r 0.36–0.48). Specifically, the Polychoric’s r of the ICECAP-A score with EQ-5D-3 L score and EQ-VAS is 0.45 and 0.42 respectively, which indicates moderate correlation between them [31].

**Table 8:** Correlation coefficient between ICECAP-A and EQ-5D-3 L

		EQ-5D-3 L					EQ- 5DL score	EQ- VAS
		Mobility	Self-care	Usual activities	Pain/discomfort	Anxiety/depressed		
ICECAP-A	Stability	0.39	0.37	0.31	0.44	0.51	0.39^a^	0.35^a^
	Attachment	0.35	0.35	0.29	0.39	0.43	0.34^a^	0.37^a^
	Autonomy	0.52	0.52	0.48	0.33	0.39	0.38^a^	0.32^a^
	Achievement	0.26	0.24	0.29	0.32	0.36	0.27^a^	0.28^a^
	Enjoyment	0.36	0.41	0.34	0.40	0.51	0.38^a^	0.37^a^
ICECAP-A score		0.38^a^	0.38^a^	0.36^a^	0.41^a^	0.48^a^	0.45	0.42

^a^Correlation coefficient were Polyserial’s r analysis. All the others are Polychoric’s r

Table 9 shows sociodemographic characteristics that correlated with the ICECAP-A and EQ-5D-3 L scores as well as each item of the ICECAP-A. The total average score for the ICECAP-A for females is statistically significant and lower than for males. Specifically, females seem to feel less stable, autonomous, fulfilled than males, but there appears to be no difference in gender in the other attributes, nor is there any for EQ-5D-3 L scores. Marital status correlates with ICECAP-A significantly. Compared to those who are single, married persons tend to have higher ICECAP-A scores, as a result of higher levels of all attributes. Being divorced or widowed is negatively correlated with ICECAP-A, especially on the attributes of stability, attachment and enjoyment. Respondents with Urban Workers’ Basic Medical Insurance have higher ICECAP-A scores for stability, autonomy and achievement compared to those with no health insurance, but not in regard to other items. Age, education, job characteristics, and income have no correlation with ICECAP-A and EQ-5D-3 L scores.

**Table 9:** Linear regression results of ICECAP-A attributes and individuals’ characteristics

Variables	ICECAP-A score	EQ-5D-3 L score	Stability	Attachment	Autonomy	Achievement	Enjoyment
Age	− 0.0008	− 0.0008	− 0.0049^a^	− 0.0045^a^	0.0011	−0.0041	− 0.0041
Gender (referent = male)							
Female	−0.0166^b^	0.0108	−0.1216^c^	− 0.0239	− 0.0921^b^	− 0.0777^a^	− 0.0402
Education (referent = high school degree or below)							
College degree	0.0033	−0.0144	−0.0453	− 0.0807	−0.024	0.0303	0.0824
Bachelor degree or above	0.0181	−0.0085	−0.0278	− 0.0622	0.0544	0.0786	0.1013
Marital status (referent = unmarried)							
Married and cohabiting	0.0318^c^	− 0.0089	0.1087^a^	0.1662^c^	0.1125^a^	0.1645^b^	0.1406^b^
Other marital status	−0.0529^b^	− 0.1424^c^	− 0.2647^b^	− 0.2125^a^	− 0.1453	−0.1409	− 0.2221^b^
Job (referent = other job)							
Business services	−0.0078	0.0126	−0.0783	0.0626	−0.0966	−0.0038	− 0.0307
Administration	0.0153	0.0230^a^	0.0632	0.1055^a^	0.0989	0.0707	0.0584
Service personnel	−0.0043	0.0123	0.019	−0.0227	0.0038	0.0624	−0.0031
Technical personnel	0.0005	0.0074	−0.0318	0.0176	0.0491	0.0326	−0.0045
Household registration (referent = non rural)							
Rural	0.0228^b^	0.0153	0.0617	0.0498	0.1315^b^	0.0708	0.0367
Health insurance (referent = Urban Resident Basic Medical Insurance)							
Full Insurance	−0.0095	0.0132	−0.0219	0.093	0.0464	−0.0147	0.1102
Urban Workers’ Basic Medical Insurance	0.0231^b^	− 0.0015	0.2025^c^	0.0712	0.1147^b^	0.1171^b^	0.0669
New Rural Cooperative Medical Insurance	−0.013	− 0.0231^a^	0.0149	−0.0134	− 0.1616^b^	− 0.0804	−0.102
Commercial Medical Insurance	0.013	0.0175^b^	− 0.016	0.0677	0.0344	0.0368	0.1252^c^
Other insurance	− 0.0891^b^	− 0.0718^a^	− 0.2407	−0.2066	− 0.0645	−0.4551^a^	− 0.5918^c^
No insurance	0.0089	0.0074	0.0467	0.0067	0.0576	−0.0044	0.033
Incomes adults	0.0047	−0.0093^b^	0.0114	0.0401^a^	− 0.033	0.0304	0.0391
Monthly income per capita (referent = <¥1000)							
¥1000 to ¥2000	0.0372	0.0765	−0.277	0.2509	0.3707	0.6163^b^	− 0.0172
¥2000 to ¥3000	−0.0052	0.0565	−0.3477	0.1715	0.1975	0.409	−0.2608
¥4000 to ¥5000	0.0172	0.0702	−0.3049	0.2195	0.2437	0.5321^a^	− 0.2066
More than ¥5000	0.016	0.0648	−0.2644	0.2102	0.2656	0.6903^b^	− 0.1849
Unknown monthly income	−0.0063	0.1478^b^	− 0.5625	0.2454	−0.0965	0.9049^b^	− 0.2777
Income source (referent = salary)							
Agriculture production or business	0.0079	−0.0544^c^	0.0225	0.0478	− 0.067	0.1309^b^	0.0347
Other income source	−0.0053	0.0102	−0.0258	− 0.0464	0.0277	0.0029	0.0607
Number of houses	0.001	0.0014^b^	0.0053	0.0032	0.0029	0.0041	0.0044

Note: reported coefficients are in the raw form

^a^significant at the 10% level

^b^significant at the 5% level

^c^significant at the 1% level

## Discussion and Conclusion

The ICECAP-A is a newly developed general QoL measurement instrument in the UK. No published study is available for its use in non-English speaking countries yet. Although there are cultural differences between English and non-English speaking countries, our study suggests that a version of ICECAP-A that slightly adapts the terminology for the two attributes is able to measure general QoL in China.

Our results show that the adapted version of ICECAP-A has good acceptability and internal reliability. As for validity, Davis et al. [29] and Keeley et al. [22] both have previously suggested two factors about “physical functioning/health” and “(psychosocial) wellbeing” [22, 32]. The principal results presented in this paper suggest that “physical health state” and “psychosocial health state” may accurately reflect the constructs termed in health economic evaluation. Therefore, our study suggests an appropriate internal validity of the ICECAP-A previous study has shown that the total score of the ICECAP-A questionnaire is significantly correlated with the total score, as well as anxiety and depression in the EQ-5D-3 L questionnaire (> 0.5). Yet, the results of our study show these two indexes have a relatively low correlation (0.45, 0.48 respectively) [19].

Keeley’s study also finds that the total score of ICECAP-A is moderately associated with that derived from EQ-5D-3 L, however their correlation index 0.49 is higher than our study 0.45. This may be attributed to our study being generalizable to general population, whereas Goranitis’ and Keeley’s focused on patients in their studies [22]. Moreover, among the 36 correlation indexes between ICECAP-A and EQ-5D-3 L, Keeley’s study shows 22 (61.11%) indexes are larger than 0.3, while our study has only 9 (25%) indexes. Although the two studies demonstrate minor differences in the results of their correlation index, they particularly show a consistency in which indexes for the dimension of anxiety/depression are generally greater than other dimensions.

Our results confirmed previous literature that demonstrated that ICECAP complements EQ-5D measures [22, 32]. Specifically, each dimension in the ICECAP is moderately associated with the items of pain/discomfort and anxiety/depressed. In contrast, the items of daily activities that may primarily represent physical healthy are relatively weakly correlated with the ICECAP score. The results in this study show that ICECAP-A was more refined for assessing well-being and the distribution of each item in ICECAP-A may suggest that the “floor effect” of EQ-5D-3 L can be avoided.

Our results indicate that ICECAP-A may be able to capture broader dimensions of QoL compared with EQ-5D. First, compared to male, females have lower ICECAP-A scores, while there is no gender difference for EQ-5D-3 L scores. As indicated by Sen, compared to males, females in many developing countries may have less capabilities in terms of their resource availability such as education and political freedom to carry out functions [33, 34].Second, compared to those with Urban Resident Basic Medical Insurance, respondents with Urban Workers’ Basic Medical Insurance have higher ICECAP-A scores. This confirmed the assumption that availability of health insurance is an important resource for capability [35].Consistent with previous literature, our results show no significant correlation between ICECAP-A and other socio-demographic variables, such as age and education [17, 19, 29]. However, unlike results from previous UK studies, our results found no significant positive relationship between income and ICECAP-A [17, 36, 37].

In China, the capability approach has been introduced recently and several measures have been developed to assess capability in inequality studies [35, 38, 39]. However, no capability approach study is available in the health economics discipline. Due to the escalating health care expenditures and overtreatment problems in China, economic evaluation has received increasing attention from policy makers, including designing drug formularies of national essential health insurance policies and medical pricing [40]. However, most of the health economic evaluations are narrowly focused on pharmaceutical products. In addition to clinical outcomes, EQ-5D is the commonly used instrument for outcome measurement [41]. Currently, the Chinese government and policy makers are putting more efforts into improving public health [42]. Due to the complexity of new public health interventions, an appropriate approach is needed to conduct the economic evaluation of these health maintaining and improving strategies [41, 42].

Our study has a few limitations. First, the online sample for the survey is composed of younger and better educated individuals than is expected in the general population. The sample size is also relatively small. Thus, we need to be cautious when generalizing these results to the general population. Further studies with large national representative sample is needed to add evidence to the international literature on the validity and use of the ICECAP-A. Second, we don’t have respondents’ objective health status in order to directly measure the correlation of EQ-5D and ICECAP with health. In the future, we will not only collect data from a larger sample with clinical health status measurement, but also apply it to patients and the elderly population to test the sensitivity of the ICECAP-A with social and healthcare interventions. Third, the ICECAP score was calculated based on the UK setting which may not reflect the value of Chinese population appropriately. It is necessary to develop a set of Chinese ICECAP values to conduct economic evaluation.

In summary, although the current study has some limitations, it represents an important first step to adapt ICECAP-A for capability well-being measurement in China. It suggests that ICECAP-A is valid and can complement the EQ-5D to measure well-being and general QoL, which can be helpful for health and social care decision-making.

## Abbreviations

EQ-5D or EQ-5D-3L: a standardized instrument for use as a measure of health outcome
HRQoL: Health-related quality of life
ICECAP-A: Investigating choice experiments capability measure for Adults
QALY: Quality adjusted life year
QoL: Quality of life
